# Impact of Wound Protectors on Complications Following Pancreaticoduodenectomy: A National Surgical Quality Improvement Program Analysis of 20 960 Patients

**DOI:** 10.1002/jso.70020

**Published:** 2025-06-30

**Authors:** Tyrell Wees, Sukhdeep Jatana, Kevin Verhoeff, A. M. James Shapiro, David L. Bigam, Khaled Dajani, Blaire Anderson

**Affiliations:** ^1^ Department of Surgery University of Alberta Edmonton Alberta Canada

## Abstract

**Background:**

Wound protectors (WPs) have been shown to decrease postoperative wound complications, yet limited data exist supporting WP for pancreaticoduodenectomies, with limited uptake in practice. We evaluated the effect of WP in pancreaticoduodenectomies on surgical site infections (SSIs) and serious complications.

**Methods:**

Utilizing the National Surgical Quality Improvement Program database, we included patients undergoing pancreaticoduodenectomy between 2017 and 2021. Baseline demographics and complications were compared between WP and no WP cohorts. Multivariate logistic regression was performed to identify the effect of WP use on 30‐day complications and factors associated with WP use.

**Results:**

Of 20 960 patients, 6167 (29.4%) used a WP. WPs were more commonly used in lower ASA classes, more comorbid patients, and preoperative weight loss. WP use was associated with increased operative time but decreased length of stay, SSIs, organ space infection, pancreatic fistula, reoperation, and serious complication. WP was independently associated with decreased serious complication (aOR 0.80, *p* < 0.001) and SSI (aOR 0.57, *p* < 0.001). Factors associated with increased likelihood of WP use include preoperative weight loss, broad‐spectrum antibiotic use, absence of bleeding disorder and firmer pancreatic texture.

**Conclusion:**

WP use during pancreaticoduodenectomy is associated with decreased number of postoperative complications and SSI. Future prospective randomized studies should assess cost–benefit and barriers to use to increase uptake of WP use.

## Summary

- This study uses the National Surgical Quality Improvement Program database to evaluate the usage of and outcomes associated with wound protectors during pancreaticoduodenectomy.
- The results showed that wound protectors are associated with a significant decrease in many 30‐day postoperative complications on multivariate analysis including surgical site infections and serious complications.
- However, uptake was limited at 29.4%, with a multivariable model showing factors such as preoperative weight loss, broad‐spectrum antibiotic use, absence of bleeding disorder, and firm pancreatic texture, associated with increased likelihood of use.

## 1 Introduction

Despite advances in surgical techniques for pancreaticoduodenectomy (PD), morbidity rates remain high. Surgical site infections (SSIs) after pancreaticoduodenectomy remain a frequent postoperative complication occurring in 6%–17% of cases [1]. SSIs can lead to prolonged length of stay (LOS), increase risk of facial dehiscence, and can prolong a patients time to adjuvant therapy and contribute a substantial burden to both minimally invasive and open PD [2, 3, 4, 5, 6, 7].

Wound protectors (WPs) act as a barrier between the intrabdominal and enteric contents and the abdominal wall in an attempt to reduce SSI. The use of WP in other abdominal surgery has been shown to significantly reduce the incidence of SSIs [8, 9]. Similarly, studies evaluating WP during PD have shown a reduced incidence of SSI. However, these studies remain limited to single‐center, small cohorts and there remains inconsistency with the adoption of this practice [10, 11, 12, 13]. Furthermore, studies have yet to assess association of WP use with other complications. Evaluating WP use during PD using a larger multicenter cohort would be beneficial to better understand its impact.

This study aims to provide the largest multicenter cohort study evaluating the impact of WP in patients undergoing PD, assessing its impact on SSI. Furthermore, it aims to expand on previous literature by assessing WP use with important outcomes such as serious complications and mortality, as well as determining factors predictive of patients receiving a WP.

## 2 Methods

### 2.1 Study Design and Population

We performed a retrospective cohort study utilizing the National Surgical Quality Improvement Program (NSQIP) database to assess the impact of WP on postoperative complications for PD patients. All patients in the NSQIP database between 2017 and 2021 who underwent PD were included using Current Procedural Terminology (CPT) codes 48152, 48153, 48150, and 48154. The NSQIP database prospectively records deinteified, standardized metrics across 702 hospitals. This data registry is regularly viewed to ensure appropriate data recording. As it is an anonymous database, ethics approval was not sought.

The primary objective of this study is to assess the impact of WP on postoperative SSI and serious complications in PD patients. Secondary objectives include assessment frequency for WP use, demographics of patients for which WP is used, and factors associated with use.

### 2.2 Outcomes and Variable Definitions

Descriptive variables collected for patient's baseline characteristics included age, gender, functional status, smoking use, and body mass index (BMI). Comorbidities that were collected include chronic obstructive pulmonary disease (COPD), congestive heart failure (CHF), hypertension (HTN), diabetes mellitus (DM), steroid use, and bleeding disorders. Other perioperative and disease factors collected include tumor invasion (≥ T3), recent weight loss, use of broad‐spectrum antibiotics, duct size, American Society of Anesthesiologists (ASA) classification, presence of preoperative sepsis, preoperative biliary stenting, and neoadjuvant therapy.

Outcomes collected include perioperative and postoperative course such as operative length, LOS in hospital, need for transfusion, need for reintubation, readmission, and death. Infectious complications collected include SSI, pneumonia, postoperative sepsis, and urinary tract infection. Surgery‐specific complications include pancreatic fistula development and the presence of wound disruption. Other complications collected include pulmonary embolism (PE), acute renal failure (ARF), cerebrovascular accident (CVA) with neurological deficit, cardiac arrest, myocardial infarction, and deep vein thrombosis (DVT) requiring treatment.

Demographic and outcome variables were defined according to the NSQIP participant user file [14]. Additionally, we evaluated 30‐day serious complications, which is a composite variable including any of the following adverse postoperative events: unplanned intubation, ARF, stroke/CVA, cardiac arrest requiring CPR, myocardial infarction, postoperative sepsis or septic shock, need for reoperation, or LOS > 30 days. POPF was collected as both a binary variable (presence or absence) and was divided into grades according to the updated International Study Group for Pancreatic Surgery (ISGPS) definition [15].

### 2.3 Statistical Analysis

Two cohorts of patients, those with WP use and those without (NWP) were compared. Categorical variables were represented with absolute numbers along with percentages while continuous variables were represented with mean and standard deviation. Comparison of categorical data and of continuous table with was done with *χ* ^2^ test and bivariate linear regression, respectively. For multivariable logistic regression models, a hypothesis‐driven purposeful selection methodology was used along with factors showing significant with *p* < 0.1 on bivariate analysis. To assess fit of model, receiver operating curves (ROC) and Brier score were used. Data analysis was performed with STATA 17 (StataCorp, College Station, TX, USA).

## 3 Results

### 3.1 Study Population Characteristics

A total of 20 960 patients met our inclusion criteria, of which 6167 (29.4%) used a WP (Table 1). There were no differences in WP and NWP cohorts in terms of demographic factors such as age, gender, BMI, functional status, and smoker status. The pathologic indication for pancreaticoduodenectomy was primarily for pancreatic ductal adenocarcinoma (53.2%). However, there were key differences in comorbidities with patients having COPD and CHF patients more likely to have WP use and those with bleeding disorders and preoperative sepsis less likely. Some disease‐specific factors also seemed to be associated with WP use, such as those with softer pancreatic gland textures, receiving neoadjuvant therapy, preoperative biliary stent, and preoperative weight loss were more likely to have WP use. Notably, larger duct size was inversely related to WP use and postoperative broad‐spectrum antibiotic use was directly associated with WP use.

**Table 1:** Baseline characteristics for patients undergoing pancreaticoduodenectomy with and without use of wound protector.

	No wound protector *n* = 14 793	Wound protector *n* = 6167	*p*
*Demographics*			
Male	7904 (53.4%)	3309 (53.5%)	0.765
Age	65.60 (11.43)	65.31 (11.37)	0.098
Body mass index	27.26 ± 5.74	27.37 ± 5.79	0.218
Smoker	2378 (16.1%)	1027 (16.7%)	0.301
Functional status			0.674
Partially dependent	120 (0.8%)	45 (0.7%)	
Totally dependent	11 (0.07%)	5 (0.08%)	
Unknown	14 (0.09%)	3 (0.05%)	
*Comorbidities*			
ASA class			*0.049*
1	35 (0.2%)	15 (0.2%)	
2	2591 (17.5%)	1140 (18.5%)	
3	10864 (73.5%)	4532 (73.5%)	
4	1295 (8.8%)	476 (7.7%)	
5	3 (0.02%)	1 (0.02%)	
Not assigned	5 (0.03%)	3 (0.05%)	
Chronic obstructive pulmonary disorder	551 (3.7%)	274 (4.4%)	*0.015*
Congestive heart failure	87 (0.6%)	62 (1.0%)	*0.001*
Hypertension	7750 (52.4%)	3285 (53.3%)	0.246
Diabetes mellitus			0.847
Noninsulin dependent	2019 (13.7%)	835 (13.5%)	
Insulin dependent	2055 (13.9%)	875 (14.2%)	
Dialysis	44 (0.3%)	24 (0.4%)	0.287
Steroid use	514 (3.5%)	186 (3.0%)	0.092
Bleeding disorder	500 (3.4%)	132 (2.1%)	*< 0.001*
*Preoperative factors*			
Preoperative sepsis			*0.004*
SIRS	163 (1.1%)	37 (0.6%)	
Sepsis	60 (0.4%)	19 (0.3%)	
Septic shock	2 (0.01%)	0 (0.00%)	
Invasion	4743 (32.8%)	1957 (32.3%)	0.475
Preoperative weight loss	1564 (13.1%)	798 (16.7%)	*< 0.001*
Diagnosis			*0.002*
PDAC	7915 (53.5%)	3234 (52.4%)	
Pancreatitis	351 (2.4%)	205 (3.3%)	
Neuroendocrine tumor	1270 (8.6%)	500 (8.1%)	
Ampullary tumor	2273 (15.4%)	1003 (16.3%)	
Pancreatic cyst	188 (1.3%)	68 (1.1%)	
Cholangiocarcinoma	780 (5.3%)	333 (5.4%)	
Other	2016 (13.6%)	824 (13.4%)	
Broad‐spectrum antibiotics	4991 (35.2%)	2604 (43.5%)	*< 0.001*
Neoadjuvant therapy	3812 (25.9%)	1758 (28.6%)	*< 0.001*
Preoperative biliary stent	7638 (54.5%)	3413 (57.1%)	*0.001*
*Intraoperative factors*			
Pancreatic gland texture			*< 0.001*
Hard	4521 (30.6%)	2380 (28.6%)	
Intermediate	1324 (9.0%)	804 (13.0%)	
Soft	4972 (33.6%)	2189 (35.5%)	
Unknown	3976 (26.9%)	794 (12.9%)	
Open vs. MIS			*0.001*
Open	13378 (90.4%)	5486 (89.0%)	
MIS	1415 (9.6%)	681 (11.0%)	
Duct size			*< 0.001*
> 6 mm	1773 (15.5%)	891 (16.0%)	
3–6 mm	5932 (52.0%)	3162 (56.7%)	
< 3 mm	3706 (32.5%)	1523 (27.3%)	

*Note:* Significant values are italicized.

Abbreviations: ASA, American Society of Anesthesiologists; SIRS, systemic inflammatory response syndrome; PDAC, pancreatic ductal adenocarcinoma; MIS, minimally invasive surgery.

### 3.2 Comparison of Complication Rate Between WP and no WP Cohorts

There were a notable number of perioperative outcomes and complications that were different between WP and NWP groups (Table 2). Operative time was longer in WP groups (382.9 min vs. 370.5 min) but duration of hospital stay was shorter (9.5 ± 6.4 days vs. 10.3 ± 6.7 days). In addition, there was less risk of multiple infections and wound complications in the WP cohort; this included a decreased risk of superficial SSI (4.9% vs. 7.5%), deep SSI (0.6% vs. 0.9%), organ space infection (15.9% vs. 18.6%), and wound disruption (0.9% vs. 1.5%). Other disease‐specific complications, such as need for transfusion and need for reoperation, were significantly less likely. Development of a clinically relevant pancreatic fistula was also less likely in the WP cohort (13.2% vs. 15.4%, *p* < 0.001), driven primarily by decreased frequency of Grade B fistulas (11.7% vs. 14.2%). The risk of 30‐day serious complications was also decreased (16.0% vs. 19.4%, *p* < 0.001) in the WP cohort, but not 30‐day mortality.

**Table 2:** Outcomes for patients who underwent pancreaticoduodenectomy with and without wound protector.

	No wound protector *n* = 14 793	Wound protector *n* = 6167	*p*
Operative time	370.5 ± 128.1	382.9 ± 132.0	*< 0.001*
Hospital length of stay (days)	10.3 ± 6.7	9.5 ± 6.4	*< 0.001*
> 30 days length of stay	501 (3.4%)	177 (2.9%)	0.054
Days from operation to discharge	9.8 ± 6.0	9.1 ± 5.9	*< 0.001*
Superficial SSI	1108 (7.5%)	303 (4.9%)	*< 0.001*
Deep SSI	132 (0.9%)	35 (0.6%)	*0.016*
Organ space infection	2751 (18.6%)	978 (15.9%)	*< 0.001*
Wound disruption	216 (1.5%)	56 (0.9%)	*< 0.001*
Pneumonia	646 (4.4%)	184 (3.0%)	*< 0.001*
Reintubation	481 (3.3%)	205 (3.3%)	0.788
Pulmonary embolism	199 (1.4%)	58 (0.9%)	*0.015*
Acute renal failure	167 (1.1%)	67 (1.1%)	0.790
Urinary tract infection	381 (2.6%)	118 (1.9%)	*0.004*
CVA with neurological deficit	34 (0.2%)	18 (0.3%)	0.411
Cardiac arrest	175 (1.2%)	82 (1.3%)	0.379
Myocardial infarction	221 (1.5%)	67 (1.1%)	*0.021*
Transfusion requirement	2962 (20.0%)	1031 (16.7%)	*< 0.001*
DVT requiring therapy	458 (3.1%)	160 (2.6%)	0.050
Sepsis	1478 (10.0%)	481 (7.8%)	*< 0.001*
Septic shock	452 (3.1%)	188 (3.0%)	0.979
Reoperation	858 (5.8%)	267 (4.3%)	*< 0.001*
Readmission	2591 (17.5%)	1021 (16.6%)	0.094
Serious complication	2875 (19.4%)	984 (16.0%)	*< 0.001*
Death	266 (1.8%)	104 (1.7%)	0.576
POPF grade			*< 0.001*
Biochemical leaks	718 (4.9%)	349 (5.7%)	
B	2080 (14.2%)	715 (11.7%)	
C	169 (1.2%)	93 (1.5%)	

*Note:* Significant values are italicized. All continuous variables are represented as mean (SD) and all categorical variables are represented as absolute values (%).

Abbreviations: CVA, cerebrovascular accident; DVT, deep venous thrombosis; m, minutes; POPF, postoperative pancreatic fistula; SSI, surgical site infection.

### 3.3 Predictors of 30‐Day Serious Complications, Mortality, and SSI

Multivariate logistic regression models were run for three outcomes including serious complications, mortality, and SSI. The model evaluating factors associated with 30‐day serious complications demonstrated that WP use was independently associated with a significant protective effect (aOR 0.80, 95% CI 0.12–0.89 *p* < 0.001, Table 3). In addition, broad‐spectrum antibiotics were also protective (aOR 0.90, 95% CI 0.82–1.00, *p* < 0.047). On the other hand, comorbidities including increased BMI (aOR 1.02, 95% CI 1.02–1.03, *p* < 0.001), COPD (aOR 1.57, 95% CI 1.26–1.96, ptable < 0.001), steroid use (aOR 1.37, 95% CI 1.05–1.80, *p* = 0.022), bleeding disorder (aOR 1.31, 95% CI 1.01–1.71, *p* = 0.044), preoperative sepsis (aOR 2.23, 95% CI 1.57–3.16, < 0.001), functional status (aOR 1.85, 95% CI 1.16–2.96, *p *= 0.010), preoperative weight loss (aOR 1.18, 95% CI 1.03–1.35, *p* = 0.018), soft pancreatic texture (aOR 1.50, 95% CI 1.46–1.66, *p* < 0.001), and preoperative biliary stent placement (aOR 1.19, 95% CI 1.07–1.31, *p* < 0.001) were associated with increased odds of 30‐day serious complications (Table 3). The Brier score and ROC for this model were 0.1432 and 0.6057, respectively. While not included in multivariate analysis due to collinearity, minimally invasive approach was found to be a protective factor for 30‐day serious complications (aOR 0.90, *p* = 0.015) on univariate analysis.

**Table 3:** Multivariable logistic regression model for factors predictive of 30‐day serious complications.

	Multivariate OR	*p*
Wound protector	0.80 (0.12–0.89)	*< 0.001*
Age	1.00 (1.00–1.01)	0.198
Body mass index	1.02 (1.02–1.03)	*< 0.001*
Female	0.77 (0.70–0.85)	*< 0.001*
Chronic obstructive pulmonary disorder	1.57 (1.26–1.96)	*< 0.001*
Congestive heart failure	1.87 (0.98–3.56)	0.056
Hypertension	1.18 (1.06–1.31)	*0.002*
Diabetes		
Noninsulin dependent	1.00 (0.87–1.16)	0.951
Insulin dependent	1.00 (0.88–1.13)	0.856
Smoker	1.09 (0.95–1.25)	0.214
Dialysis	1.44 (0.68–3.04)	*0.339*
Steroid use	1.37 (1.05–1.80)	*0.022*
Bleeding disorder	1.31 (1.01–1.71)	*0.044*
Preoperative sepsis	2.23 (1.57–3.16)	*< 0.001*
Functional status	1.85 (1.16–2.96)	*0.010*
Preoperative weight loss	1.18 (1.03–1.35)	*0.018*
Invasion (≥ T3)	1.04 (0.94–1.15)	0.409
Neoadjuvant	0.96 (0.86–1.08)	0.499
Soft pancreas	1.50 (1.46–1.66)	*< 0.001*
Preoperative biliary stent	1.19 (1.07–1.31)	*0.001*
Broad‐spectrum antibiotics	0.90 (0.82–1.00)	*0.047*

*Note:* Significant *p* values are italicized. Values presented as odds ratio (95% confidence interval). Brier score: 0.1432, ROC area under curve: 0.6057.

Evaluating 30‐day mortality, WP use was not independently associated with any significant change (aOR 1.03, 95% CI 0.76–1.40, *p* = 0.833, Table 4). Comorbidities tended to increase risk with the highest odds with dialysis‐dependence, poor functional status, CHF, and COPD. Soft pancreas texture and preoperative weight loss were also associated with increased odds of mortality. The Brier score and ROC were 0.0163 and 0.6700, respectively, indicating a good fit.

**Table 4:** Multivariable logistic regression model for factors predictive of 30‐day mortality.

	Multivariate OR	*p*
Wound protector	1.03 (0.76–1.40)	0.833
Age	1.03 (1.02–1.05)	*< 0.001*
Body mass index	1.04 (1.02–1.07)	*0.001*
Female	0.84 (0.63–1.13)	0.253
Chronic obstructive pulmonary disorder	1.78 (1.03–3.05)	*0.037*
Congestive heart failure	4.11 (1.41–11.96)	*0.009*
Hypertension	1.04 (0.76–1.42)	0.827
Diabetes		
Insulin dependent	0.95 (0.62–1.48)	0.841
Noninsulin dependent	1.27 (0.87–1.87)	0.211
Smoker	1.33 (0.89–1.98)	0.166
Dialysis	2.64 (0.61–11.44)	0.195
Steroid use	1.96 (1.02–3.76)	*0.044*
Bleeding disorder	1.84 (0.98–3.45)	0.059
Preoperative sepsis	1.29 (0.46–3.59)	0.626
Functional status	2.05 (0.73–5.79)	0.174
Preoperative weight loss	1.77 (1.24–2.52)	*0.002*
Invasion (≥ T3)	1.13 (0.84–1.53)	0.420
Neoadjuvant	1.03 (0.73–1.46)	0.880
Soft pancreas	1.39 (1.03–1.88)	*0.033*
Preoperative stent	1.03 (0.76–1.39)	0.865
Broad‐spectrum antibiotics	0.83 (0.61–1.12)	0.212

*Note:* Significant *p* values are italicized. Values presented as odds ratio (95% confidence interval). Brier score: 0.0131, ROC area under curve: 0.6700.

Finally, WP use was independently associated with decreased likelihood of SSI (both superficial and deep) (aOR 0.57, 95% CI 0.47–0.69, *p* < 0.001, Table 5). Similarly, broad‐spectrum antibiotic use was associated with decreased odds (aOR 0.69, 95% CI 0.58–0.82, *p* < 0.001). Demographic factors such as female sex and higher BMI were also independently associated with SSI but notably many comorbidities included in the analysis were not (Table 5). The Brier score and ROC are 0.0196 and 0.5339, respectively.

**Table 5:** Multivariable logistic regression model for factors predictive of 30‐day surgical site infection, superficial or deep.

	Multivariate OR	*p*
Wound protector	0.57 (0.47–0.69)	*< 0.001*
Age	1.00 (0.99–1.00)	0.309
Body mass index	1.03 (1.01–1.04)	*< 0.001*
Female	1.23 (1.04–1.45)	*0.012*
Chronic obstructive pulmonary disease	0.98 (0.63–1.52)	0.938
Congestive heart failure	0.80 (0.19–3.39)	0.766
Hypertension	1.08 (0.91–1.29)	0.363
Diabetes		
Noninsulin dependent	0.87 (0.68–1.12)	0.287
Insulin dependent	1.06 (0.84–1.34)	0.608
Smoker	1.16 (0.93–1.45)	0.200
Dialysis	1.14 (0.26–4.94)	0.860
Steroid use	1.26 (0.80–1.99)	0.325
Bleeding disorder	0.95 (0.59–1.54)	0.840
Preoperative sepsis	0.64 (0.28–1.46)	0.283
Functional status	0.94 (0.37–2.38)	0.901
Invasive disease (≥ T3)	1.00 (0.85–1.18)	0.984
Neoadjuvant	0.98 (0.82–1.18)	0.856
Soft pancreas	1.21 (1.02–1.43)	*0.028*
Preoperative stent	1.83 (1.52–2.20)	*< 0.001*
Preoperative weight loss	0.92 (0.73–1.16)	0.462
Broad‐spectrum antibiotics	0.69 (0.58–0.82)	*< 0.001*

*Note:* Significant *p* values are italicized. Values presented as odds ratio (95% confidence interval). Brier score: 0.1432, receiver‐operating curve area under curve: 0.6054.

### 3.4 Subgroup Analysis Evaluating High‐Risk and Low‐Risk Patients With WP Use

Furthermore, subgroup analysis evaluated WP use in both high‐risk and low‐risk groups. High‐risk patients were defined as those with a soft pancreas, male sex, with diabetes, smokers, and those receiving steroids or neoadjuvant therapy. In high‐risk patients (*n* = 13 706), WP use remained independently associated with a reduction in serious complications (aOR = 0.79, *p* < 0.001) and SSI (aOR = 0.64, *p* < 0.001). Similarly, in low‐risk patients (*n* = 1869), WP use was independently associated with reduced likelihood of SSIs (aOR = 0.51, *p* = 0.006).

### 3.5 Patient Factors Associated With WP Use

On multivariate logistic regression, factors identified to be associated with WP use included the presence of preoperative weight loss, broad‐spectrum antibiotic, and neoadjuvant chemotherapy, which increased likelihood of WP use (aOR 1.32, *p* < 0.001, aOR 1.43, *p* < 0.001, and aOR 1.13, *p* = 0.003, respectively; Table 6). The presence of a bleeding disorder and preoperative sepsis (aOR 0.56, *p* < 0.001 and aOR 0.54, *p* = 0.001) was associated with decreased use. The Brier score and ROC are 0.02056 and 0.5740, respectively.

## 4 Discussion

This is the largest study to date evaluating WP use in PD and demonstrates that WP use was associated with decreased rates of SSI and certain postoperative complications, including on multivariate analysis. Furthermore, we demonstrate that WP use results in improved outcomes for both high‐risk and low‐risk patients. Despite these results, WP is used in less than one‐third of PD.

Results from this study are in keeping with previous studies which demonstrated an OR of 0.59 for the development of SSI with the use of WP use in general surgery cases [8]. Furthermore, previous smaller studies evaluating WP use in PD have demonstrated a odds ratio of 0.70 reduction in SSI [10]. Overall, our findings are in keeping with previous studies, with an odds ratio of 0.57, but add to the literature as the largest cohort study which also controls for potential confounders such as antibiotic use, preoperative biliary stenting, and pancreatic gland texture using multivariable modeling. In addition to association with 30‐day SSI, this study also demonstrated that WP was independently associated with a reduced likelihood of serious complication. While this has not been explored in previous studies evaluating WP use in PD it may be due to decreased contamination, trauma, and bacterial exposure to the superficial tissues. Reduced SSI can lead to decreased rates of sepsis, fascial dehiscence (and need for reoperation), and LOS, which are likely the key drivers for the effect on serious complications [3]. Furthermore, despite WP use being shown to decrease organ space infections (18.6% vs. 15.9%, *p* < 0.001), a significant driver for these infections remains pancreatic fistula formation [16, 17]. Existing evidence demonstrates decreased POPF development and infection rate with use of broad‐spectrum antibiotics [16, 17, 18]. As demonstrated in Table 5, broad‐spectrum antibiotic use was independently associated with decreased 30‐day SSI (aOR 0.69, *p* < 0.001), further adding to the existing evidence supporting broad‐spectrum antibiotic use to decrease SSI rates. In terms of our secondary outcome, multivariable analysis demonstrated that use of WP was less likely in patients with known bleeding disorder, preoperative sepsis, or soft pancreatic texture. This may be due to theoretical increased trauma to the tissues which may provoke bleeding or the theoretical decreased likelihood of malignant seeding in patients with soft pancreatic texture. Factors associated with increased use include postoperative broad‐spectrum antibiotic use which may be part of center‐specific initiatives to decrease SSI through multimodal approaches. Additionally, perceived higher risk populations such as patients with diabetes, dialysis, steroid use, or with tobacco use were not shown to have increased usage of WPs. However, our data support the use of WP being an independent factor in reducing SSI in both high‐ and low‐risk populations. Furthermore, we have demonstrated that WP is also protective against 30‐day serious complications in high‐risk patients. This would support broad use of WP in most PD patients, barring contraindications, regardless of their perceived risk. Additionally, the large variability in WP use, and lower scores of predictive tests such as ROC and Brier score, further support that there are no set guidelines on which patients should have WP used intraoperatively. Due to the limitations of the database, it is difficult to assess center‐to‐center practices (as some centers may not use them at all while others use it routinely) and implications of cost and insurance status on these results.

**Table 6:** Multivariable logistic regression model for factors predicting the use of WP during PD.

	Multivariate OR	*p*
Age	1.00 (0.99–1.00)	0.069
Body mass index	1.01 (1.00–1.01)	0.107
Female	1.00 (0.93–1.07)	0.895
Chronic obstructive pulmonary disease	1.22 (1.02–1.46)	0.027
Congestive heart failure	1.23 (0.72–2.10)	0.454
Hypertension	1.05 (0.97–1.14)	0.204
Diabetes		
Noninsulin dependent	0.98 (0.88–1.09)	0.751
Insulin dependent	0.99 (0.89–1.10)	0.872
Smoker	1.03 (0.93–1.13)	0.609
Dialysis	1.27 (0.70–2.30)	0.606
Steroid use	0.87 (0.70–1.08)	0.435
Bleeding disorder	0.56 (0.44–0.71)	*< 0.001*
Preoperative sepsis	0.54 (0.37–0.77)	*0.001*
Functional status	0.97 (0.65–1.45)	0.870
Invasive disease (≥ T3)	0.97 (0.90–1.05)	0.418
Neoadjuvant	1.13 (1.04–1.23)	*0.003*
Preoperative stent	1.05 (0.98–1.13)	0.186
Preoperative weight loss	1.32 (1.20–1.46)	*< 0.001*
Broad‐spectrum antibiotics	1.43 (1.33–1.54)	*< 0.001*

*Note:* Significant *p* values are italicized. Values presented as odds ratio (95% confidence interval). Brier score: 0.2056, receiver‐operating curve area under the curve: 0.5740.

Despite evidence to support the use of WP both in abdominal surgery and specifically PD to reduce SSI, the uptake, as evidenced by this study, remains low. In our NSQIP analysis, less than one‐third of PDs were performed with WP. However, the proportion of WPs being used during PD was shown to increase between 2017 and 20217 (26.6% and 32.6%, *p* < 0.001). One potential hypothesis explaining the slow uptake for WPs may be the associated cost or environmental impact. Previous studies evaluating the cost of WPs in appendectomy or colorectal surgery have demonstrated no significant cost saving [19, 20]. However, given the relatively high rate of SSI in PD patients (superficial SSI 6.7%%, deep SSI 0.8%, organ space infection 17.8%), the high‐risk patient population, preoperative weight loss and malnutrition, and the risk of delayed recovery given extent of surgery, we hypothesize that WP may reduce costs in PD. Future studies and RCTs evaluating WP in this patient population are encouraged to also conduct cost and environmental analyses to completely understand the role of WPs in PD.

Limitations of this study include that this is a retrospective cohort study, which prevents determination of causality, and due to the patient data being collected from a database, there are limitations to which variables are collected, which prevents subsequent analyses. The NSQIP database does not allow for individual surgeon outcomes and experience. We were also not able to look at individual centers which may have adopted the use of WPs during PD. Another limitation is that we were not able to assess the individual type of WP used (e.g., single ring vs. double ring).

Despite the limitations, this study is the largest study assessing the impact of WP use during PD. It supports the use of WP in PD in both univariate and multivariate analysis to reduce the risk of SSI as well as serious complications postoperatively. Future studies should include multicenter prospective cohort study or randomized control trial to objectively assess WP effects on post‐PD complications as well as cost–benefit analyses to show the impact on costs and feasibility.

## 5 Conclusion

This study demonstrates that WP use during PD is independently associated with reduced likelihood of SSI and serious complications. However, WP use remains limited and randomized studies to confirm these results and economic analysis to understand the cost of WPs are needed. Future studies should assess barriers to implementation, and can include prospective cost–benefit analyses, as well as identifying populations who may benefit most from WP use.

## Conflicts of Interest

The authors declare no conflicts of interest.

## Synopsis

This study uses the National Surgical Quality Improvement Program database to evaluate the usage of and outcomes associated with wound protectors during pancreaticoduodenectomy. The results showed that wound protectors are associated with a significant decrease in many 30‐day postoperative complications on multivariate analysis including surgical site infections and serious complications. However, uptake was limited at 29.4%, with a multivariable model showing factors such as preoperative weight loss, broad‐spectrum antibiotic use, absence of bleeding disorder, and firm pancreatic texture, associated with increased likelihood of use.

## Data Availability

The data that support the findings of this study are available from the corresponding author upon reasonable request.
